# Immunohistochemical Study Using Monoclonal VE1 Antibody Can Substitute the Molecular Tests for Apprehension of BRAF V600E Mutation in Patients with Non-small-Cell Lung Carcinoma

**DOI:** 10.1155/2019/2315673

**Published:** 2019-11-05

**Authors:** Hadeel Abdul Elah Karbel, Sura Salman Ejam, Ali Zaki Naji

**Affiliations:** ^1^Pathology Dept. Hammurabi College of Medicine, University of Babylon, Iraq; ^2^Pathology Dept. College of Medicine, University of Babylon, Iraq; ^3^Basic Science Dept. College of Dentistry, Babylon University, Iraq

## Abstract

In patients with non-small-cell lung carcinoma (NSCLC), the analysis of BRAF V600E mutation has become more and more applied since the introduction of many mutation-targeted medications. In this regard, the advantage of immunohistochemistry (IHC) as a reliable diagnostic test substitute to other molecular studies has not been approved yet. *Objective.* To examine the dependability of using immunohistochemical method utilizing monoclonal VE1 antibody in the detection of BRAF V600 E mutation in patients with non-small-cell lung carcinoma and compare the results there with that of polymerase chain reaction (SSCP-PCR). *Materials and Methods*. We retrospectively identified 53 patients of whom their histopathological diagnosis was non-small-cell carcinoma of different types. Evaluation of BRAF V600E mutation was assessed using polymerase chain reaction (SSCP-PCR) and IHC using VE1 antibody. This approach was applied to all cases under the study. *Results*. Among the 53 NSCLC samples, only 5 (9.3%) cases harbored BRAF V600E mutation, 80% were of adenocarcinoma type, and the rest (20%) was of squamous cell carcinoma. IHC analysis for VE1 was positive in 4 out of 5 (80%) BRAF-mutated tumors and negative in all nonmutated BRAF V600 E NSCLC. *Conclusion*. Our results revealed that VE1 antibody IHC analysis is a promising technique that can be used to detect BRAF V600-mutated NSCLC with relatively high specificity and sensitivity and might become a potential alternative to the current molecular biological methods that are in use for this purpose.

## 1. Introduction

For considerable decades, lung cancer has been considered the major outstanding cause of cancer-related mortality globally [1].

The GLOBOCAN database revealed that lung cancer is responsible for about 19% of all cancer-causing death worldwide in 2019 [2].

Broadly, the WHO classification (2015) of tumors of the lung, pleura, thymus, and heart has been subclassified into two major types of the epithelial tumors of the lung: small-cell lung cancer (SCLC) which accounts for 15% of lung carcinomas and non-small-cell lung cancers (NSCLs) which account for the 85% remainder of all lung carcinomas [3, 4]. Almost two-thirds of those patients are diagnosed at progressive stages of the disease; thus, their therapeutic options are limited with poor prognosis and low survival rate [5, 6]. However, the overall survival of such patients with advanced disease stage can be enhanced by rapid administration of target-specific drugs against sure genetic changes in either EGFR or ALK genes. Furthermore, several therapeutics target specific somatic mutations involving different oncogenes [7, 8], like v-raf murine sarcoma viral oncogene homolog B (BRAF) mutation. These mutations will play vital role in predicting patient outcome and response to target medications [9, 10].

The BRAF codes for a nonreceptor serine/threonine kinase, in which the latter is an important member of the RAS/RAF/MEK mitogen-activated protein kinase (MAPK). Mutation in BRAF would subsequently result in pathway alteration and sustained kinase activity which are one of the corner stones in the process of carcinogenesis [11, 12].

The bulk of these mutations are caused by hotspot transversion mutation at exon 15, which leads to amino acid substitution of V600 E [13].

BRAF V600E mutation has been established in various types of cancers, like melanoma, papillary thyroid carcinoma, and metastatic colorectal adenocarcinoma with a frequency of mutation at about 50%, 45%, and 9%, respectively [14, 15]. While previous studies have shown that the prevalence of BRAF mutation in lung carcinoma is approximately 2-4% [8, 10, 16].

The diagnosis of lung carcinoma is frequently achieved in small-sized biopsies, obtained either by bronchoscopy or CT-guided/echo-guided routes, thus dealing with such small biopsies must be optimized in order to reach the final diagnosis taking into consideration the current ancillary techniques which can be performed in FFPE tissue biopsies that allow both histopathological and immunohistochemical features to be characterized, as well as analysis and extraction of DNA for further molecular studies [17].

In this study, we aimed to study the pervasiveness of BRAF V600E mutation in biopsies of NSCLC patients using SSCP-PCR in comparison to immunohistochemical study for the same gene.

## 2. Materials and Methods

This is a retrospective study carried out from December 2017 to March 2019. We include patients with NSCLC from several private labs and Teeba Respiratory Center in Hilla city, Babylon province. The analytic data of 53 patients with NSCLC were retrieved; however; we could not obtain all the clinical data for some patients like the stage of the disease; formalin-fixed paraffin-embedded (FFPE) tissue samples for those patients were also collected; all the results were reviewed by three expert histopathologists and final confirmation of the diagnosis was done. While in cases of poorly differentiated tumors, we needed to use ancillary IHC to reach the final diagnosis. A similar number (53) of normal lung tissues was also included and used as control samples for the PCR study.

Ethical clearance was attained from the Scientific committee of the Hamourabi College of Medicine, University of Babylon.

## 3. Molecular Study

### 3.1. FFPE Tissue DNA Extraction

FFPE tissue section samples were extracted using NEXprep™ FFPE Tissue Kit, Genes Laboratories, Korea. The extraction was achieved according to the manufacturer's protocol. In brief, xylene was used to remove the paraffin waxes and was washed out by absolute ethanol. Then, genomic DNA was extracted with Proteinase K, and aliquots from the extracted DNA samples were quantified using a NanoDrop spectrophotometer. Finally, the samples were placed in -20°C for further use in SSCP-PCR experiments.

### 3.2. Single-Strand Conformation Polymorphism (SSCP)

SSCP-PCR was performed for detection of V600E mutation in exon 15 of the BRAF gene from the lung carcinoma and normal lung tissue samples. The method was carried out according to Kobayashi et al. [18]. The BRAF gene exon 15 primers include forward primers (5′-CCTAAACTCT TCATAATGCTTGCTC-3) and reverse primer (5′-TTAATCAGTGGAAAAAT AGCCTCAA-3) provided by Macrogen, Korea. The PCR master mix was prepared according to the user manual. (AccuPower® PCR Pre Mix kit, Bioneer, Korea). The PCR tubes contain a pellet consisting of (Taq DNA polymerase 1U, Tris-HCl (pH 9.0) 10mM, KCl 30mM, stabilizer, MgCl 2 1.5mM, dNTPs 250*μ*M, and tracking dye). The preparation of the master mix was achieved according to the protocol provided by the kit. The PCR assay was achieved to a final reaction volume of 20 *μ*l consisting of 5 *μ*l of DNA ,1.5 *μ*l of 10 pmol of each forward primer and reverse primer mixed together, and then the volume was completed to 20 *μ*l with deionized water. The reaction mixture was then mixed, briefly vortexed, and placed in the thermocycler (T100 Thermal cycler, Bio-Rad, USA) with the subsequent thermal conditions:

1. Initial denaturation temperature was 94°C for 5 min
2. 35 cycles at denaturation 94°C for 30 s
3. Annealing was achieved at 58°C for 30 s
4. Extension was 72°C for 1 min
5. Final extension was 72°C for 5 min

After that, the SSCP for BRAF mutations were done by denaturing PCR products through incubation at 95°C for 6 min; then, the specimens were immediately placed on ice. After that, the quality and quantity of PCR amplicons were then confirmed with 1% agarose gel electrophoresis with subsequent visualization by UV illumination.

### 3.3. Immunohistochemistry

**Figure 1:**
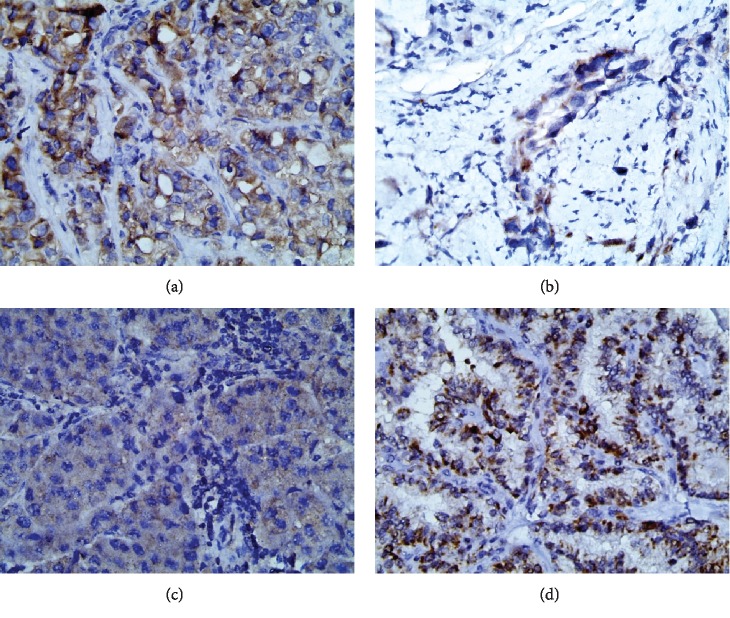
Immunohistochemical pathological examination of NSCLC using VE1 antibody. (a) Poorly differentiated adenocarcinoma with strong cytoplasmic staining (score+3) ×200. (b) Poorly differentiated adenocarcinoma with moderate cytoplasmic staining (score+2) ×200. (c) Moderately differentiated adenocarcinoma with weak cytoplasmic staining (score+1) ×200. (d). Positive control papillary carcinoma of the thyroid with strong cytoplasmic staining ×200.

The clinical specimens used were surgically resected biopsies (*n* = 38) and bronchoscopically obtained biopsies (*n* = 15). From each tissue block, two sections were stained with hematoxylin and eosin (H and E) method and immunohistochemical polydetector plus horseradish peroxidase staining method using monoclonal mouse antihuman BRAF V600 protein, ready-to-use, Bio SB, Clone L50-823, USA. A tumor was considered positive for V600E immunostaining when uniform signal was detected in the cytoplasm of at least 50% of the tumor cells and the intensity scoring graded as zero (negative), +1(weak cytoplasmic signal), +2(moderate cytoplasmic signal), and +3(strong cytoplasmic signal) according to Sasaki et al. [19]. Positive control from papillary thyroid carcinoma tissues was concerned in every run.  Figure 1(d).

### 3.4. Statistical Analysis

Statistical analysis was accomplished using SPSS version 20. Absolute variables were presented as frequencies and percentages. A *p* value of equal or less than 0.05 was selected as a significant value.

## 4. Results

**Figure 2:**
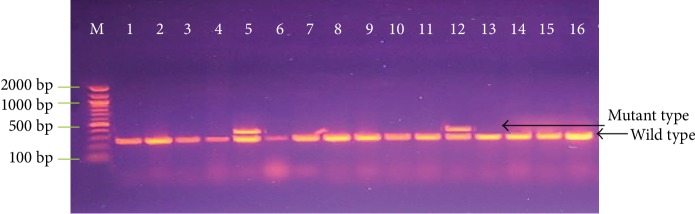
Agarose gel electrophoresis image that show the SSCP-PCR product analysis for the detection of V600E mutation in exon 15 of BRAF gene from NSCLC and normal lung tissue samples where M is marker (2000-100 bp), lanes 1-4, 6-11, and 14-16 are wild-type, and where lanes 5 and 12 are V600E mutation.

The clinical aspects for patients with NSCLC involved in this study is summarized in Table 1. Out of 53 cases of NSCLC, 5 (9.3%) were shown to have BRAF V600E mutation in exon 15 in comparison to 53 samples of normal lung tissues which revealed only the wild type of the gene by using SSCP-PCR (Figure 2).

The particular clinical features for patients with mutant BRAF are mentioned in Table 2. Regarding the histology of NSCLC with mutant BRAF gene, 4 cases (80%) were adenocarcinoma, while only one case (20%) was squamous cell carcinoma.

BRAF wild-type gene was detected in 48 (90.6%) of patients' samples with NSCLC, 30 were males, 18 were females with age ranging between 45 and 85 years.

There was no important association between BRAF wild-type and mutant cases concerning the gender, age, and histopathological types of NSCLC (*p* > 0.05).

An immunohistochemical study with VE1 monoclonal antibodies revealed positive results in 4 (80%) out of 5 cases with mutant BRAF V600E as demonstrated by SSCP-PCR. Furthermore, our results show a significant association between IHC results and PCR results for the detection of BRAF V600E mutational status in patients with NSCLC (*p* value = 0.0001) with a chi‐square value = 41.535.  Table 3.

Comparison of IHC results with that of PCR study, declared sensitivity of 97.9% and specificity of 100%.

The immunohistochemical results were reviewed and validated by three pathologists with 100% concordances which were considered positive if homogenous intracytoplasmic staining was shown in carcinoma cells solely. The intensity of immunohistochemical results was scored from 1-3 consequently, with no significant association with the type of the tumors and degree of differentiation (*p* > 0.05) (Figure 1(a)-(c)).

## 5. Discussion

The central objective of this study was to explore the possibility of utilizing monoclonal VE1 antibody immunohistochemical test as a surrogate for the presently used molecular techniques in the detection of BRAF V600E mutation in NSCLC patients. Toward this, we tend to start with testing molecular mutation of BRAF V600E utilizing SSPC-PCR technique. We found BRAF V600E mutations in 9.3% of the patients; this is often comparatively higher than those reported in other series (0.8%-4.9%) [10, 16, 18, 20, 21, 22], whereas a study carried out by Ilie et al. [17] revealed 9% BRAF mutation which is nearly similar to our results. It is worth mentioning that their study was achieved in EGFR, K RAS, PI3KCA, HER2, and EML4-ALK wild-type adenocarcinoma only. Nonetheless, we included all types of NSCLC in our samples and only 50.9% were of adenocarcinoma type.

Similar to other studies [10, 16, 23], BRAF V600E mutation is more prevalent in adenocarcinoma type (80%) with exceptionally one case (20%) of squamous cell carcinoma. Most of these cases were of high-grade, poorly differentiated solid type, and this could explain the relatively higher frequency of BRAF mutation in our study. These results are similar to Yousem et al.'s [24] and Kobayashi et al.'s [18]. findings which demonstrated that the majority of BRAF-mutated NSCLC was of high grade and poor prognosis. Chen et al. [20] in their systemic review and meta-analysis for patients with mutant BRAF NSCLC found that Asians have a somewhat higher tendency of harboring BRAF alteration than others; however, this association is weak and of no statistical significance.

Our results that there have been no vital association between prevalence of BRAF mutation and the patient gender (*p* > 0.05) is incontestable, with similar results demonstrated by Cardarella et al. [25], Ilie et al. [17], and Chen et al. [20] Such association was previously observed in female patients with BRAF mutant colorectal carcinoma [26, 27].

Then, we went on emulating BRAF mutation using IHC methodology. We noted that VE1 monoclonal antibody achieved high concurrence rates with the molecular practice (*p* < 0.05). Similar concordance pattern was reported by Ilie et al. [17], Sasaki et al. [19], and Gow et al. [28] who found that IHC with VE1 clone is a very sensitive and specific method for the detection of mutant BRAF gene in lung adenocarcinoma. Likewise, Luk et al. [21] demonstrated that BRAF IHC was positive in two out of three cases with V600E gene alteration, and their results were steady with the Sequenom massARRAY platform results.

Indeed, immunohistochemical analysis for VE1 mutation was recommended as a predictable methodology for detection of BRAF V600E mutation in alternative tumors like melanoma [29], papillary carcinoma of the thyroid [30, 31], and colorectal carcinoma [32].

Given the very fact that BRAF V600E mutational status is clinically of great prognostic value, determination of this mutation has become increasingly performed as an adjunct to histopathological study, since there are many BRAF pathway-targeting agents in clinical advancement and trials, such as XL281, selumetinib, and PLX4032 [33, 34].

For BRAF gene mutation in NSCLC detection, analysis was carried out by utilizing molecular methods including DNA extraction from FFPE biopsies [35], such methods can lead to depletion of tissue samples or the samples themselves are already not sufficient for such molecular approach because most of the biopsies that had been used in the diagnosis are bronchial or core needle transthoracic specimens. Consequently, the adoption of another specific and sensitive method for the detection of BRAF V600E gene mutation on these tissue sections may grant the conservation of the samples and also provide low-charge procedure.

Several drawback points were also reported in the molecular analytic techniques [36, 37], used for BRAF gene mutation in metastatic brain tumors. These techniques revealed negative results as compared to IHC which was capable of identifying a small portion of BRAF V600E-expressing carcinoma cells. Such inconsistency was interpreted by Ilie et al. [38] due to hyper fixation of DNA, existence of necrotic tumor areas, or low frequency of BRAF-mutated cells, which in turn lead to a decrease in efficiency of molecular techniques for identification of BRAF mutation. In our study, only one case was IHC negative for VE1, and this could be due to several factors including improper tissue fixation and heterogenous expression of the antigen which could be overcome by staining multiple sections from the same tissue samples; however, this is not always possible in small tissue biopsies.

Katerina et al. [30] in their study for the foremost impact preanalytical conditions for the IHC detection of BRAF V600E (VE1) antibody on colorectal and papillary thyroid carcinoma conclude that the most proper tissue fixation ought to be done within 2 hours of tissue collection for 12-24 hours in 10% neutral buffered formalin.

## 6. Conclusion

The current study provides new data concerning BRAF immunohistochemical technique as a reliable methodology for the analysis of the mutational status of BRAF V600E in NSCLC patients especially with the recent development of mutation-specific BRAFV600E monoclonal antibodies which made it a rapid and cost-effective test for those patients.

## Figures and Tables

**Table 1:** Clinicopathological characteristics of patients with non-small-cell lung carcinoma (no. = 53).

	No. (%)
Gender	
Male	30 (56.6%)
Female	23 (43.4%)
Age	
45-55	12(22.6%)
56-65	19(35.9%)
66-75	17(32.1%)
76-85	5(9.4%)
Histological types	
ADENO CA	27(50.9%)
SQUAMOUS CA	22(41.5%)
ADENO/SQ CA	2(3.8%)
Large cell CA	2(3.8%)
Differentiation	
Well	12(22.6%)
Moderately	23 (43.3%)
Poorly	18(33.9%)

ADENO CA: adenocarcinoma; SQUAMOUS CA, squamous cell carcinoma: ADENO/SQ CA, adeno-squamous carcinoma.

**Table 2:** Clinicopathological characteristics of patients with mutant BRAF V600E tumors (no. = 5).

Patient	BRAF mutation	IHC VE1	IHC intensity scoring	Age	Sex	Histological type	Differentiation
1	Positive	Positive	+3	61	M	Adeno Ca	Poorly diff.
2	Positive	Positive	+1	65	F	Adeno Ca	Poorly diff.
3	Positive	Negative	Zero	62	M	Squamous Ca	Moderate diff.
4	Positive	Positive	+2	59	M	Adeno Ca	Poorly diff.
5	Positive	Positive	+1	67	F	Adeno Ca	Moderate diff.

F, female; M, male; adeno Ca, adenocarcinoma; squamous Ca, squamous carcinoma; diff, differentiated.

**Table 3:** The association of IHC of BRAF VE in patients of NSCLC with mutant type BRAF V600E by SSCP-PCR.

		IHC BRAF VE1		Total
		Positive	Negative	
BRAF V600E SSCP-PCR	Mutant gene	4 (7.5%)	1 (1.9%)	5 (9.4%)
	Wild gene	0(0%)	48(90.6%)	48 (90.6%)
Total		4 (7.5%)	49 (92.5%)	53(100%)

*p* value = 0.0001. Chi‐square value = 41.535.

## Data Availability

The data findings of this research have to be seen in light of some restrictions including the small sample size of the study group, retrospective design of the study, and lack of patient follow-up for detection of the survival rate for those with mutant BRAF V600E NSCLC.
